# Formation of ecocardiografia in critics' area with malingerer VIMEDIX

**DOI:** 10.1186/2036-7902-4-S1-A10

**Published:** 2012-12-18

**Authors:** Raul Manuel Vicho Pereira

**Affiliations:** 1 Clinica USP-Palmaplanas, Medicina Intensiva, Palma de Mallorca, Spain

## 

We realize a transthoracic (TTE) and transesophageal echocardiography (TOE) course for Anesthesia Department specialized in resuscitation with periopertative care cardiac surgery. The teacher was Raul Vicho, echocardiography teacher accredited by the Spanish Society of Intensive Medicine and Coronary Units (SEMICYUC).

## Objective

To demonstrate if a theoretical - practical TTE and TOE course with the malingerer VIMEDIX 10 days, it was sufficient to acquire a level for the exercise of being technical in this service.

## Method

The duration of the course was 10 days in view of theoretical classes (40 hours) and 60 hours of simulation (TOE and TTE with 40 pathological suppositions) and transthoracic studies in patients for 4 Anesthetists combining TTE and TOE probe. The theoretical program was:

1) Basic concepts of planes and doppler.

2) Hemodinamics ventricular right function.

3) Pericardium

4) Acute Aortic Syndrome

5) Image Planes

6) Valvulart heart disease

7) Masses, tumors and endocarditis.

The studies in patients have been completed by a report written for the area of the critical patient checked by the teacher. At the end of the course they realized an examination of 30 representative questions of the formation type test, evaluation practises of acquisition of planes of TTE and TOE and 10 clinical representative cases.

## Results

The results of the examination was > 75 % corrects answers, > 8 corrects cases and they acquired > 80 % of planes of TTE and TOE. They manage to acquire in 7 minutes 15 TOE standars. They did TTE report of sufficient quality to continue doing it in the patients' area.

## Conclusion

A course given by a teacher of the SEMICYUC with malingerer VIMEDIX of 10 days is equivalent to the formation of a supporting level IIb of the SEMICYUC in TTE of the critical patient or to the advaced of European Company of Intensive Medicine. TOE basic level qualifies them to realize basic studies without issuing reports.

